# RBE‐weighted dose and its impact on the risk of acute coronary event for breast cancer patients treated with intensity modulated proton therapy

**DOI:** 10.1002/acm2.13527

**Published:** 2022-01-21

**Authors:** Chunbo Liu, Julie A. Bradley, Dandan Zheng, Raymond B. Mailhot Vega, Chris J. Beltran, Nancy Mendenhall, Xiaoying Liang

**Affiliations:** ^1^ University of Florida Health Proton Therapy Institute Jacksonville Florida USA; ^2^ Department of Radiation Oncology University of Florida College of Medicine Jacksonville Florida USA; ^3^ Department of Radiation Oncology University of Nebraska Medical Center Omaha Nebraska USA; ^4^ Mayo Clinic Department of Radiation Oncology Jacksonville Florida USA

**Keywords:** acute coronary event, breast cancer, intensity‐modulated proton therapy, RBE‐weighted dose

## Abstract

**Purpose:**

To evaluate the relative biological effectiveness (RBE)‐weighted dose to the heart and to estimate RBE uncertainties when assuming a constant RBE of 1.1, for breast cancer patients receiving intensity‐modulated proton therapy (IMPT). Further, to study the impact of RBE uncertainties on the risk of an acute coronary event (ACE).

**Material and methods:**

We analyzed 20 patients who received IMPT to either the left breast (n = 10) or left chest wall (n = 10) and regional lymph nodes. The Monte Carlo simulation engine, MCsquare, was used to simulate the dose‐averaged linear energy transfer (LETd) map. The RBE‐weighted dose to the heart and its substructures was calculated using three different RBE models. The risk of ACE was estimated per its linear relationship with mean heart dose (MHD) as established by Darby et al.

**Results:**

The median MHD increased from 1.33 GyRBE assuming an RBE of 1.1 to 1.64, 1.87, and 1.99 GyRBE when using the RBE‐weighted dose models. The median values (and ranges) of the excess absolute risk of ACE were 0.4% (0.1%–0.8%) when assuming an RBE of 1.1, and 0.6% (0.2%–1.0%), 0.6% (0.2%–1.1%), and 0.7% (0.2%–1.1%) with the RBE‐weighted models. For our patient cohort, the maximum excess absolute risk of ACE increased by 0.3% with the RBE‐weighted doses compared to the constant RBE of 1.1, reaching an excess absolute ACE risk of 1.1%. The interpatient LETd variation was small for the relevant high‐dose regions of the heart.

**Conclusion:**

All three RBE models predicted a higher biological dose compared to the clinical standard dose assuming a constant RBE of 1.1. An underestimation of the biological dose results in underestimation of the ACE risk. Analyzing the voxel‐by‐voxel biological dose and the LET map alongside clinical outcomes is warranted in the development of a more accurate normal‐tissue complication probability model.

## 1 INTRODUCTION

According to the American Cancer Society,^1^ breast cancer is one of the most common cancers in American women, and the second leading cause of cancer death among women. For decades, radiotherapy has been part of the standard of care for breast cancer. Studies^2^, ^3^, ^4^, ^5^ have demonstrated that radiotherapy confers significant advantages in disease control and improved survival for breast cancer patients. However, radiotherapy has also been associated with increased cardiovascular morbidity and mortality because of incidental radiation to the heart.^6^, ^7^, ^8^, ^9^, ^10^ According to a study published in *The Lancet* by Darby et al.,^6^ the rate of major coronary events increases linearly with an increase in mean heart dose (MHD) by 7.4% per Gray. For radiotherapy treatment to locally advanced breast cancer involving the regional lymph nodes, the incidental MHD can reach 28.6 Gy.^11^ Such incidental radiation to the heart can detrimentally affect a patient's quality of life and compromise the survival gain of radiotherapy. Advancements in cancer care have vastly improved life expectancy among breast cancer survivors; therefore, efforts to reduce radiation‐induced toxicity have drawn increased attention.^12^ One advancement that offers a reduction in the deposit of incidental radiation is proton therapy.

Protons travel a finite distance into tissue and then release most of their energy in a tightly defined region called the Bragg peak. Due to this unique energy absorption profile, proton therapy offers the advantage of minimizing the cardiac radiation dose in breast cancer treatment.^13^, ^14^ In recent years, the number of proton facilities worldwide has increased dramatically from 27 in 2010 to 100 in 2021,^15^ and an increasing number of patients have received proton therapy for their breast cancer.^16^, ^17^ A currently enrolling multicenter pragmatic randomized clinical trial, RadComp,^18^ is aimed at assessing level I evidence of the effectiveness of proton versus photon therapy in reducing major cardiovascular events. Yet alongside the advantages of proton therapy there exist unique challenges, too, such as setup, range, and relative biological effectiveness (RBE) uncertainties. Without proper accounting for these uncertainties could negatively impact clinical outcomes and prevent full exploitation of the advantages of proton therapy. In current clinical practice, setup and range uncertainties are addressed through robust optimization,^19^, ^20^ which optimizes the cost function both on the nominal scenarios and on a set of simulated predefined setup‐error and range‐error scenarios. Our previous study^21^ demonstrated the effectiveness of robust optimization for breast proton therapy. While understanding RBE uncertainty has become an active area of research, the implications of this research have not been realized in routine clinical practice, and a constant RBE of 1.1 remains the standard practice. This constant RBE of 1.1 value was deduced based on in vivo measurements in the early days of proton therapy and has been agreed upon within the community to ensure an adequate dose is delivered to the tumor.^22^ Nevertheless, emerging data^22^, ^23^, ^24^ show that proton RBE varies, increasing when protons decelerate at the distal end and producing a biological range shift of a few millimeters beyond the physical range. Study^25^ has shown that it is more problematic for the organs at risk (OARs) immediately distal to the target due to the RBE end‐of‐range effects. In breast cancer treatment, en‐face beams are used and, therefore, the heart is located immediately distal to the clinical target volume (CTV). If the RBE uncertainty is not properly accounted for, there is a high risk of underestimating the biological dose and hence underestimating the toxicity to the heart. To better assess the risk of heart toxicity and establish a more accurate clinical outcome model, it is essential to account for the variable RBE and estimate the actual biological dose delivered to the heart.

## 2 MATERIALS AND METHODS

This study was approved by our institution's institutional review board (IRB#20170251). In the current study, two phenomenological RBE models and one linear‐fit RBE model were selected to calculate the voxel‐by‐voxel RBE‐weighted dose to the heart for patients who received intensity‐modulated proton therapy (IMPT) for their breast cancer treatment. We chose multiple RBE models to evaluate the biological dose because large variations were observed in different RBE model predictions, especially in tissue with low α/β and high linear energy transfer (LET) values.^26^ Late‐responding heart tissue immediately distal to the target that experiences the end‐of‐range LET elevation falls into this category. Therefore, to assess the impact of RBE model uncertainties and study variations in different RBE model predictions, we chose three different RBE models for the current study. The difference between the standard clinical practice of assuming a constant 1.1 RBE dose (Dose_1.1) and the RBE‐weighted doses based on various RBE models was assessed. The risk of an acute coronary event (ACE) was estimated based on the prediction model developed by Darby et al.^6^

### 2.1 Patient selection

We retrospectively reviewed the radiation treatment plans of 20 patients treated with IMPT to the regional lymph nodes and the left breast (n = 10) or left chest wall (n = 10). The CTV structures included breast tissue limited anteriorly 5 mm from the skin or chest wall limited anteriorly 3 mm from the skin, internal mammary nodes (IMN), axillary level I‐III nodes (AxI‐III), and supraclavicular nodes (SCV). OAR structures—including the heart, ventricles, left anterior descending artery (LAD), left lung, right lung, esophagus, thyroid, and brachial plexus—were also contoured. The total prescription dose delivered was 50 GyRBE (RBE = 1.1) over 25 fractions using two en‐face beams using RayStation (RaySearch Laboratories, Sweden) treatment planning system (TPS); the planning technique is detailed in our previous publication.^27^ Briefly, A 7.4‐cm water‐equivalent Lucite range shifter was used for each beam. Robust optimization was performed with 5 mm setup uncertainty and 3.5% range uncertainty. Both the optimization and dose computation used the Monte Carlo algorithm, with a 2‐mm calculation grid. The final dose was computed using the MC algorithm with 0.5% statistical uncertainty.

### 2.2 LET simulation

Because the clinical TPS does not provide the LET information, an open‐source Monte Carlo (MC) engine for proton pencil‐beam scanning (PBS), MCsquare,^28^, ^29^ was used to simulate the dose‐averaged LET (LETd). The PBS energy layers, spots geometry and weighting, and monitor units of the clinical plans were exported to MCsquare. The physical dose (PD) was calculated and the voxel‐by‐voxel dose‐averaged LET (LETd) was simulated. The statistical uncertainty of the MC simulation was set to 0.5%. The LET was scored for primary protons, secondary protons, and secondary alphas. To reduce the statistical noise of the low‐dose voxels, LET was not scored for voxels below 10 cGy. MCsquare calculation accuracy was benchmarked by comparing it with the clinical treatment planning system.

### 2.3 RBE‐weighted dose calculation

To consider the variable RBE effect, numerous models have been developed.^30^, ^31^, ^32^, ^33^ McNamara et al.^34^ and Rørvik et al.^35^ conducted comprehensive reviews on various RBE models. Most are phenomenological, based on the LETd and α/β of the linear‐quadratic model:

(1) $$\begin{array}{ccc} & & RBE\;[D_{p\;},\;\left(\alpha /\beta ){}_{x},LETd\right]\\ & & \;=\frac{1}{2D_{p}}\;\left(\sqrt{{\left(\alpha /\beta \right)}_{x}^{2}+\;4D_{p\;}{\left(\alpha /\beta \right)}_{x}\;RBE_{max}+\;4D_{p}^{2}\;RBE_{min}^{2}-{\left(\alpha /\beta \right)}_{x\;}\;}\right) \end{array}$$

where $RBE_{max}$ and $RBE_{min}$ correspond to the asymptotic value of RBE at doses 0 and ∞ Gy, respectively.^31^

In the current study, we chose two representative phenomenological RBE models: the McNamara model^32^ and the Wedenberg model.^33^ The McNamara model is based on multiple cell lines and 287 experimental data points, which consists of four parameters with LETd and α/β dependence in the quadratic dose term:

(2) $$\;RBE_{max}=\;0.99064+\;\frac{0.35605\;Gy\;{\left(Kev\;\mu m\right)}^{-1}}{{\left(\alpha /\beta \right)}_{x}}\;LETd$$

(3) $$\begin{array}{ccc} \;RBE_{min} & = & \;1.1012-0.0038703\;Gy^{-\frac{1}{2}}\;\;{\left(Kev\;\mu m\right)}^{-1}\\ & & \times \;\sqrt{{\left(\alpha /\beta \right)}_{x\;}\;}\;LETd \end{array}$$

The Wedenberg model was derived from multiple cell lines (19 experimental data points); it uses one model parameter and has no variation with the LETd or α/β of the quadratic dose term:

(4) $$\;RBE_{max}=\;1+\;\frac{0.434\;Gy\;{\left(Kev\;\mu m\right)}^{-1}}{{\left(\alpha /\beta \right)}_{x}}\;LETd$$

(5) $$\;RBE_{min}=\;1$$

Recently, simple linear‐fit models were also developed for proton RBE by obtaining the coefficient via fitting of clonogenic cell survival data.^36^, ^37^ In the current study, we also included the linear‐fit model developed by McMahon et al.^36^:

(6) RBE=1+0.055×LETd

For each patient, four doses were calculated using an α/β of 3 Gy for the heart^38^:

1. Dose_1.1: The constant RBE of 1.1 was applied. The Dose(RBE = 1.1) dose distributions were compared with the clinical plans to benchmark the MCsquare accuracy.
2. Dose_McM: The RBE‐weighted dose was calculated using the McMahon linear model.
3. Dose_McN: The RBE‐weighted dose was calculated using the McNamara model.
4. Dose_Wed: The RBE‐weighted dose was calculated using the Wedenberg model.

The differences between the standard clinical practice of assuming a constant RBE of 1.1 dose (Dose_1.1) and the RBE‐weighted doses based on various RBE models were assessed.

### 2.4 ACE risk assessment

The excess risk of the ACE due to the increased RBE‐weighted dose to the heart was estimated using the Darby models.^6^ In the study by Darby et al.,^6^ it was found that rates of ACE increased linearly with the MHD by 7.4% per Gray (95% confidence interval, 2.9 to 14.5) with no apparent threshold. The increase started within the first 5 years after radiotherapy and continued into the third decade after radiotherapy. The risk of ACE was estimated for 50‐year‐old women with no preexisting cardiac risk factors who received radiotherapy for breast cancer and attained an age of 80 years:

(7) $$4.5\%\;\left(1+7.4\%\;*MHD\right)$$

where 4.5% is the risk of ACE in the absence of RT with an attained age of 80 years, and MHD is in Gy.

### 2.5 Statistical analysis

A nonparametric Wilcoxon signed‐rank test was used for statistical analysis, with *p* < 0.05 taken as statistically significant.

## 3 RESULTS

### 3.1 Benchmark MCsquare simulation

**FIGURE 1:**
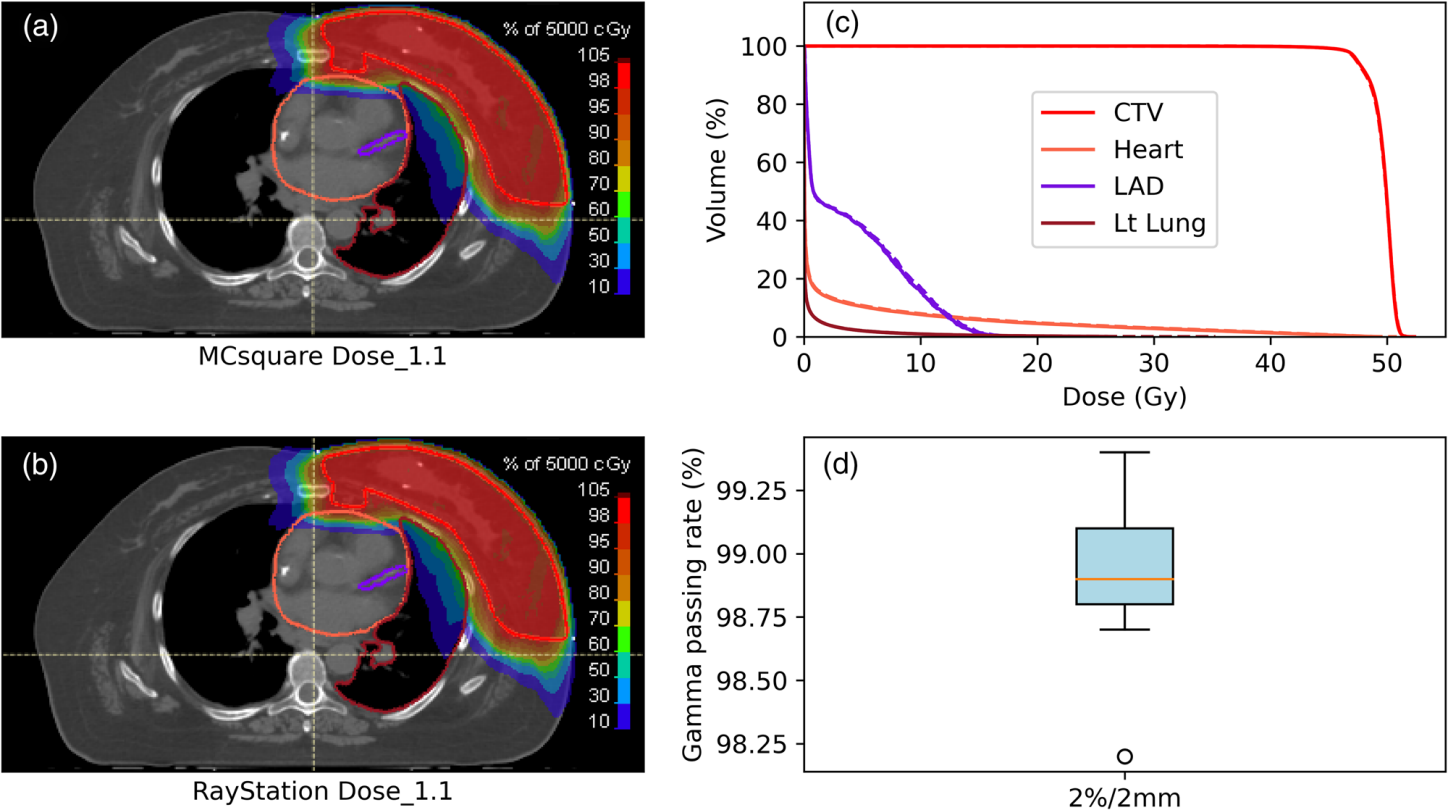
Color‐washed dose distribution of an example patient: (a) Dose_1.1 from the MCsquare calculation, (b) clinical plan, (c) DVH comparison of these two plans, and (d) box plot of the 3D gamma testing passing rate with 2%/2mmm criteria for all the studied patients

To benchmark the MCsquare simulation, the Dose_1.1 distribution from the MCsquare calculation was compared with the clinical treatment plan. 3D Gamma analysis with 2%/2 mm criteria was conducted for each patient. Excellent agreement between the MCsquare calculations and the clinical plans was observed. The 2%/2 mm gamma passing rate ranged from 98.7% to 99.4%. The MHD calculated using MCsquare was also compared with the Raystation dose. The median and range of MHD were 1.36 (0.41–2.56) GyRBE and 1.33(0.39–2.44) GyRBE from RayStation and MCsquare, respectively. Figure 1 shows (a) the dose distribution of Dose_1.1, (b) the dose distribution of the clinical plan, and (c) a dose‐volume histogram (DVH) comparison of these two plans. Figure 1d shows a box plot of the gamma passing rate with 2%/2 mm criteria for all studied patients.

In this study, the evaluation of the constant 1.1 RBE dose and RBE‐weighted doses were all based on the MCsquare simulations.

### 3.2 RBE‐weighted dose and LETd

**FIGURE 2:**
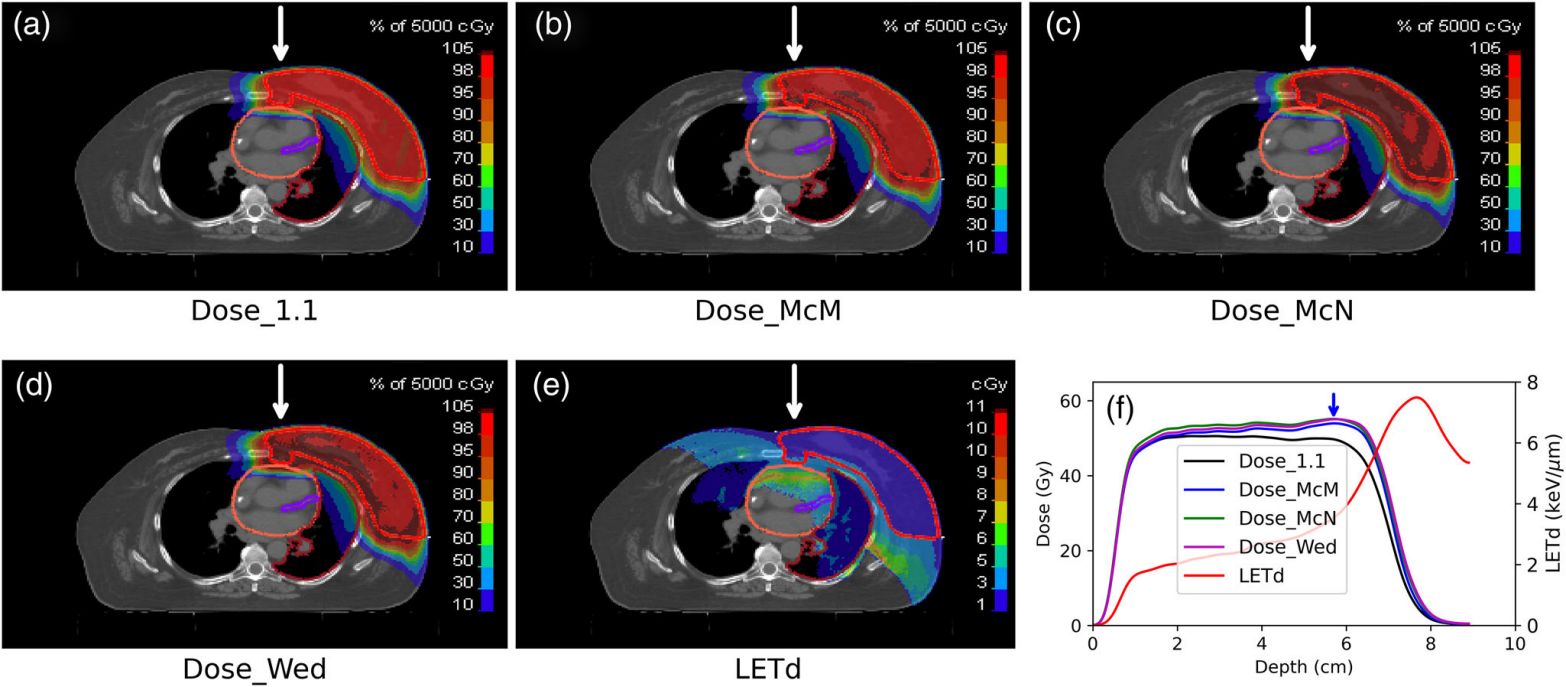
Color‐washed dose distribution of an example patient: (a) Dose_1.1, (b) Dose_McM, (c) Dose_McN, and (d) Dose_Wed. The breast CTV, heart, and ipsilateral lung contours are also shown. Figure 2(e) shows the dose‐averaged linear energy transfer (LETd) distribution. The dose profile comparison, as well as the LETd profile, are displayed in Figure 2f; the blue arrow indicates the interface of the clinical target volume and heart. The white arrow in Figures 2a–d indicates the direction of the profile drawing

**FIGURE 3:**
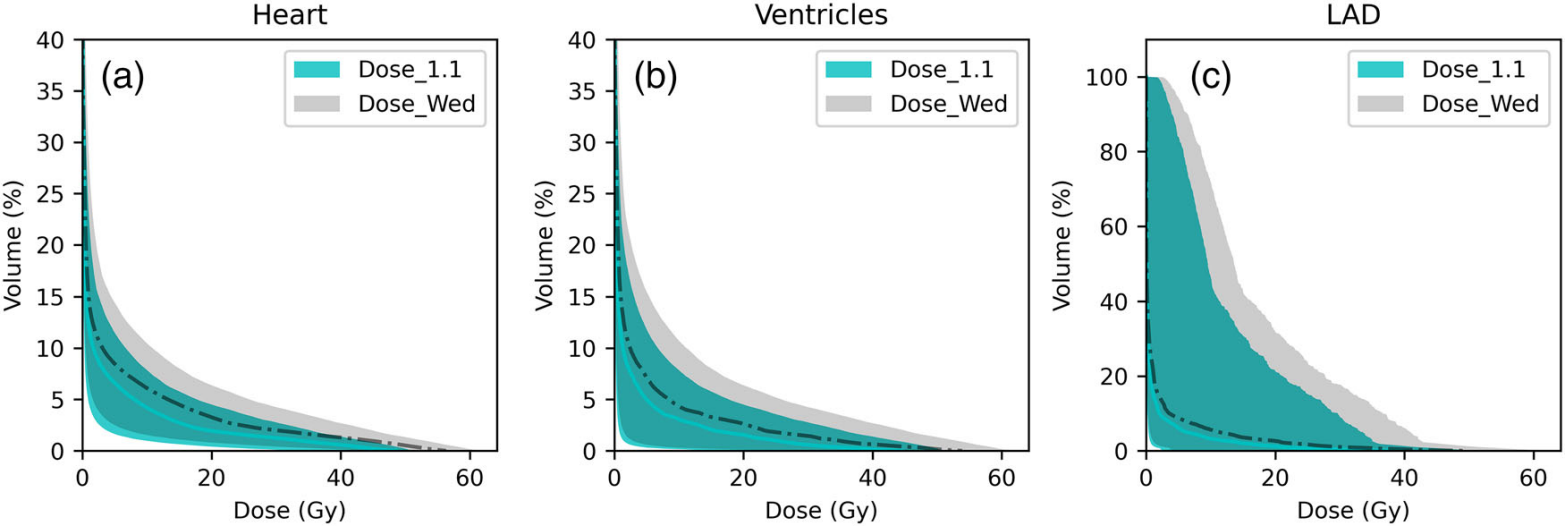
Dose‐volume histogram (DVH) of Dose_1.1 and Dose_Wed, from all patients, on the (a) heart, (b) ventricles, and (c) left anterior descending artery. The shaded region represents the range for all patients. The solid and dashed lines represent the population median DVH of the Dose_1.1 and Dose_Wed, respectively

To evaluate the impact of the variable RBE on the modeled biological dose received by the heart, the RBE‐weighted doses calculated using different RBE models were compared with the standard clinical practice of Dose_1.1. Figure 2 shows the color‐washed dose distribution, the LET distribution, as well as the LETd profile of an example patient. All three RBE models predicted a higher RBE‐weighted dose compared to the constant 1.1 RBE dose. The increase of the biological dose gets larger as it approaches the distal end of the target, and the largest enhancement was ∼2 mm beyond the CTV, which landed right in the heart. Within the CTV region, the LETd increased from ∼2 KeV/μm to ∼4 KeV/μm. Once in the heart, the LETd rapidly increased to ∼8 KeV/μm. The increased LETd, in turn, yielded a larger biological dose to the heart than what the clinical plan predicted, as shown in Table 1 and Figure 3.

**TABLE 1:** Doses statistics to the heart, ventricles, and LAD and the excess absolute risk of a coronary event

		**Dose_1.1**		**Dose_McM**		**Dose_McN**		**Dose_Wed**	
	**Patient Group**	**Median**	**Range**	**Median**	**Range**	**Median**	**Range**	**Median**	**Range**
**Heart V5 (%)**	All Patients	6.7	1.7–11.6	7.5	2.1–12.8	8.3	2.5–14.1	8.6	2.6–14.5
	CW	6.0	4.0–11.6	6.8	4.5–12.8	7.5	5.0–13.9	7.8	5.2–14.3
	Breast	6.9	1.7–11.5	7.9	2.1–12.7	8.9	2.5–14.1	9.2	2.6–14.5
**Heart V25 (%)**	All Patients	1.7	0.3–3.7	2.1	0.5–4.5	2.3	0.6–5.0	2.5	0.7–5.3
	CW	1.8	0.8–3.3	2.3	1.2–4.1	2.6	1.4–4.5	2.8	1.6–4.8
	Breast	1.4	0.3–3.7	1.9	0.5–4.5	2.2	0.6–5.0	2.4	0.7–5.3
**Mean Heart Dose (GyRBE)**	All Patients	1.33	0.39–2.44	1.64	0.50–2.92	1.87	0.58–3.25	1.99	0.62–3.41
	CW	1.32	0.95–2.41	1.65	1.15–2.89	1.84	1.31–3.22	1.94	1.39–3.38
	Breast	1.33	0.39–2.44	1.64	0.50–2.92	1.87	0.58–3.25	1.99	0.62–3.41
Ventricles V5 (%)	All Patients	4.5	0.4–11.9	5.4	0.5–13.4	6.3	0.6–15.0	6.6	0.7–15.6
LAD D0.1cc (GyRBE)	All Patients	14.84	1.83–34.16	18.16	2.23–39.20	20.56	2.82–40.94	21.72	3.05–41.81
LAD mean dose (GyRBE)	All Patients	3.85	0.44–12.06	4.78	0.51–13.96	5.73	0.64–15.44	6.16	0.69–16.11
**Excess absolute risk of ACE** **°** **(%)**	All Patients	0.4	0.1–0.8	0.6	0.2–1.0	0.6	0.2–1.1	0.7	0.2–1.1
	CW	0.4	0.3–0.8	0.6	0.4–1.0	0.6	0.4–1.1	0.6	0.5–1.1
	Breast	0.4	0.1–0.8	0.5	0.2–1.0	0.6	0.2–1.1	0.7	0.2–1.1

Abbreviations: Dose_1.1, the constant RBE of 1.1 was applied; Dose_McM, the RBE‐weighted dose was calculated using the McMahon linear model; Dose_McN, the RBE‐weighted dose was calculated using the McNamara model; Dose_Wed, the RBE‐weighted dose was calculated using the Wedenberg model.; LAD, left anterior descending artery.

^°^ The excess absolute risk of ACE is calculated for women by the age of 80 who received radiotherapy at age 50 with no preexisting cardiac risk factors based on Darby model^6^ ^.^

The DVH of Dose_1.1 and Dose_Wed on the heart, ventricles, and LAD for all 20 patients are shown in Figure 3. A clear DVH shift to the right (higher value) was observed in the RBE‐weighted dose compared to that assuming a constant RBE of 1.1. The upper boundary of the DVH band exhibited a larger shift than the lower boundary of the DVH band. Hence, a larger increase in the RBE‐weighted dose compared to the RBE of 1.1 dose was observed for the patients who received a higher physical dose. For patients who received the minimal physical dose, the increase in RBE‐weighted dose was small. Table 1 shows doses to the heart, ventricles, and LAD. Statistically significant differences (*p* value < 0.01) were observed between the Dose_1.1 and the RBE‐weighted dose for all studied dose metrics across the three RBE models. DVH metrics for all three RBE models predicted higher RBE‐weighted doses than those assuming a constant RBE of 1.1. In the current study, we also grouped patients by intact breast (n = 10) and chest wall (n = 10). The heart dose metrics and excess absolute risk of ACE of each group were also presented in Table 1. No statistical difference (*p*‐value > 0.61) was observed between these two groups. Thus, our findings are relevant across all 20 patients.

**FIGURE 4:**
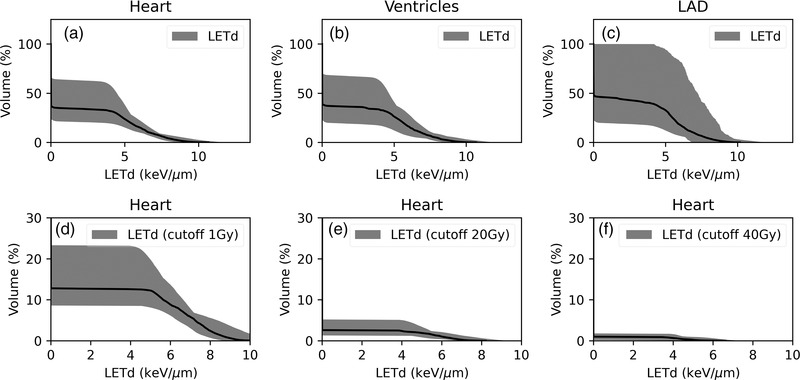
Dose‐averaged linear energy transfer (LETd) histogram on (a) heart, (b) ventricles, and (c) LAD. The heart LETd histogram with 1 Gy, 20 Gy, and 40 Gy cutoff are plotted in (d), (e), and (f), respectively. The solid line represents the population median

The LETd interpatient variations were also studied. Figure 4 plots the LETd histogram for the 20 patients on the (a) heart, (b) ventricles, and (c) LAD. LETd interpatient variation within the heart and ventricles was small for the high‐LETd regions. The greatest interpatient variations were observed for the LAD, owing to its small volume making it sensitive to interpatient location and plan quality variations. In addition, we also studied LETd with different dose cutoffs for the heart, as the high LETd does not affect voxels that receive a little dose. We plotted the LETd histogram for the heart voxels that received >1 Gy (Figure 4d), >20 Gy (Figure 4e), and >40 Gy (Figure 4f). Interpatient LETd variation decreased further within the high‐dose region.

### 3.3 Acute coronary event assessment

**FIGURE 5:**
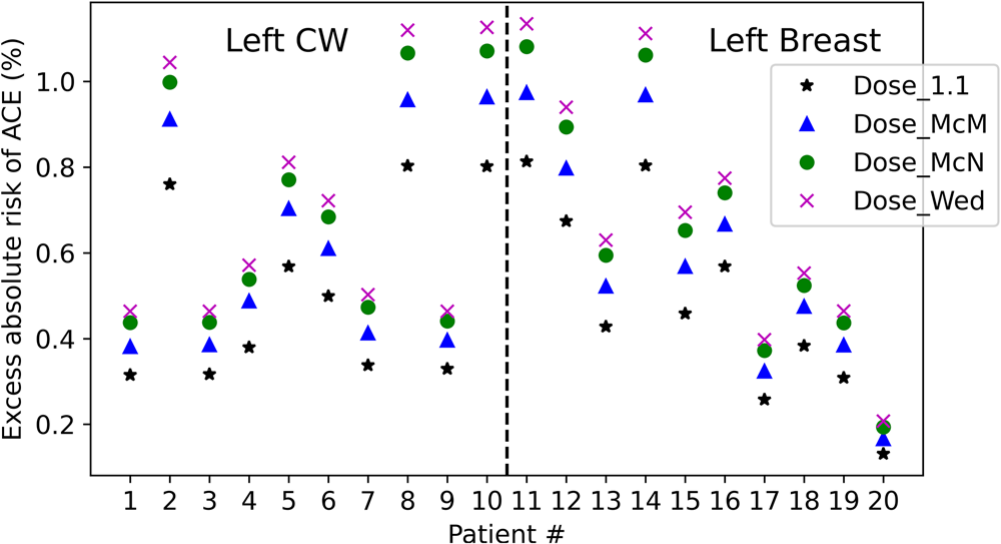
Estimation of excess absolute risk of acute cardiac events. Patients #1–10 are left CW patients and #11–20 are left, breast patients

The impact of the RBE‐weighted dose on the risk of ACE was also evaluated using the Darby model.^6^ Per the Darby model, the risk of ACE increases by 7.4% for every 1 Gy of MHD. For 50‐year‐old women with no preexisting cardiac risk factors, the absolute risk of having at least one ACE with an attained age of 80 with no radiation is 4.5%. The risk increased to 4.9% due to IMPT breast radiation based on the median MHD of 1.33 GyRBE (RBE = 1.1) of our cohort. When calculating the dose with more accurate variable RBE models, the median values of the MHD increased to 1.64 GyRBE (Dose_McM), 1.87 GyRBE (Dose_McN), and 1.99 GyRBE (Dose_Wed), corresponding to an absolute ACE risk of 5.0%, 5.1%, and 5.2%, respectively. Figure 5 plots the excess absolute risk of ACE due to radiation. The RBE‐weighted dose predicted a higher excess absolute risk of ACE compared to that predicted with a constant RBE of 1.1. No obvious difference was observed between the left CW group and the left breast group. Assuming a constant RBE of 1.1, the excess absolute risk of ACE in our study cohort was 0.8% for patients #8, 10, 11, and 14. The excess absolute risk of ACE reached 1.1% with the Wedenberg model prediction for these four patients, 0.3% higher than that based on the dose with a constant RBE of 1.1. The median values (and ranges) of the excess absolute risk of ACE for a 50‐year‐old woman with no preexisting cardiac risk factors who receive radiotherapy, and with an attained age of 80 years, were 0.4% (0.1%–0.8%), 0.6% (0.2%–1.0%), 0.6% (0.2%–1.1%) and 0.7% (0.2%–1.1%) assuming a constant RBE of 1.1, and using the McMahon, McNamara, and Wedenberg RBE models, respectively (Table 1). The RBE = 1.1 underestimates the biological dose to the heart, thereby underestimating the risk of ACE.

## 4 DISCUSSION

Cardiovascular morbidity is one of the most concerning side effects of radiation therapy for breast cancer. Darby et al.^6^ demonstrated a linear increase in the rate of major cardiovascular events with increasing MHD. The increase starts within the first 5 years after radiotherapy and continues into the third decade after radiotherapy. Proton therapy, with its sharp dose fall‐off at the distal end, provides a unique advantage by reducing the incident dose to the heart compared to photon therapy, especially for the treatment of left‐sided breast/chest wall cancer with IMN involvement. However, according to Darby et al.,^6^ there is no threshold or “safe dose” below which the risk of coronary events will not increase. Even small heart doses are suspected to increase the risk of cardiac disease. Therefore, the end‐of‐range RBE effect needs to be considered to estimate the actual biological dose delivered to the heart and to assess the risk of heart toxicity. Understanding the biological dose based on variable RBE is also essential to establishing more accurate clinical outcome models. The collection of voxel‐by‐voxel biological doses and LET maps on the heart and its substructures will provide important information for developing a more accurate normal tissue complication probability (NTCP) model.

In the current investigation, we studied 10 patients with left breast and 10 with left chest wall tumors. Patients also received regional lymph node irradiation to the IMN, AX I‐III, and SCV. The voxel‐by‐voxel LET was simulated using the MC engine, MCsquare. RBE‐weighted doses were calculated using three different RBE models (two phenomenological RBE models and one linear‐fit RBE model). An α/β of 3 Gy^38^ was used for the late‐responding heart. The potential impact of the RBE‐weighted dose compared to that with the clinical standard constant RBE of 1.1 was investigated. In addition to the dose to the heart, studies^39^, ^40^ have also shown that the incident dose to the ventricles and LAD are strongly correlated with the risk of cardiotoxicity and its severity. Therefore, in the current study, we evaluated the RBE‐weighted dose to the ventricles and LAD as well as the heart dose. The risk of ACE was estimated using the model developed by Darby et al.^6^ In our study, a clear shift of the DVH to the higher values was observed in the RBE‐weighted dose compared with a constant RBE of 1.1 across all patients and for all studied DVH metrics. The median value of the MHD increased from 1.33 GyRBE (Dose_1.1) to 1.64, 1.87, and 1.99 GyRBE when using the McMahon, McNamara, and Wedenberg RBE models, respectively. Although the RBE‐weighted dose increased from that with a constant RBE of 1.1, the MHD from proton therapy is still much lower than the overall average MHD of 3.6 Gy from a review^41^ of publications from 2014 to 2017 and MHD of 8.4 Gy from a review^11^ on publications from 2003 to 2013 analyzing photon techniques. Investigators recently analyzed the potential impact of variable RBE on the dosimetric advantage of proton therapy compared to photon techniques for breast cancer.^42^ As expected and consistent with our findings, the conclusion was that proton therapy offers advantages over photon therapy when analyzing the NTCP using dosimetric data, but the advantages decrease when applying a more accurate RBE‐weighted dose. In an analysis of the RBE‐weighted dose to heart for breast proton therapy by Oden et al.,^40^ investigators evaluated three breast plans. They found that the MHD increased from 0.18 GyRBE (RBE = 1.1) to 0.32 GyRBE (variable RBE). Since the study by Oden et al.^43^ was on the whole breast with no regional lymph nodes involvement, it is unsurprising that the MHD they reported was lower than among our study cohort. Furthermore, lymph nodes involvement greatly impacts spot placement relative to the heart. Therefore, the end‐of‐range effect on the heart can vary greatly making a comparison of their study to ours difficult.

In our study, we also observed that a higher physical dose was associated with a larger RBE‐weighted dose increase compared to the constant RBE of 1.1. For patients who received the minimal physical dose, the increase in RBE‐weighted dose was small since the increase depends on both the LET and the physical dose. For very low physical dose voxels, the RBE‐weighted dose increase was irrelevant even with a very high LET. Likewise, the excess absolute risk prediction based on the RBE‐weighted dose resembled that predicted per the Dose_1.1 for patients who received the minimum heart dose. In contrast, in patients who received relatively high heart doses, such as patients #8, 10, 11, and 14 (Figure 5), the excess absolute risk prediction based on the RBE‐weighted dose was clearly higher than that predicted based on the Dose_1.1. For these four patients, the excess absolute risk of ACE increased by 0.3% based on the RBE‐weighted doses compared with that based on a constant RBE of 1.1, reaching an excess absolute ACE risk of 1.1%. Interpatient LETd histogram variation was small for the relevant high‐dose region in the heart. The geometry of the heart and IMN are similar across patients; thus, spot placement and the high‐dose volume of the heart are likewise similar.

Notably, RBE models depend on model‐specific fitting parameters, α/β ratio, dose, end point as well as intrinsic radiosensitivity of the tissue, which can translate into potentially large uncertainties in predicted RBE.^44^ Investigators^34^, ^35^ have shown vast variations on the calculated RBE among different models. In addition, uncertainty is inherent to the Darby model as well. Therefore, it is more appropriate to interpret the findings of the current study as a relative evaluation rather than providing absolute expected values. In acknowledging the uncertainties of the RBE models, in the current study, we chose three different RBE models (two phenomenological and one linear‐fit) to calculate the RBE‐weighted doses. Our findings show that, although some variations exist among the results from the different RBE models, the constant RBE of 1.1 consistently underestimates the biological dose. Therefore, despite these uncertainties, it is safe to conclude that the constant RBE of 1.1 underestimates the dose to the patient and, hence, the risk of ACE.

## 5 CONCLUSION

Using three different RBE models, RBE‐weighted doses to the heart, ventricles, and LAD were studied for 20 patients with left breast or left chest wall tumors. Across the cohort, all three RBE models predicted higher biological doses compared to the standard clinical practice assuming a constant RBE of 1.1. The impact of the RBE‐weighted dose on the risk of ACE was assessed per the Darby model. The excess absolute risk based on the RBE‐weighted dose reached 0.3% higher than that determined with a constant RBE of 1.1. An accounting of voxel‐by‐voxel biological dose alongside the LET map and clinical outcome data are warranted to develop a more accurate NTCP model.

## AUTHOR CONTRIBUTIONS

XL developed the concept. CL carried out the date collection. XL and CL conducted the study with the help from all other authors. XL drafted the manuscript. All the authors provided clinical expertise and participated in writing the manuscript. All authors read and approved the final manuscript.

## CONFLICT OF INTEREST

The authors declare no conflict of interest.

## Data Availability

The data that support the findings of this study are available from the corresponding author upon reasonable request.
